# Overview of MicroRNAs in Cardiac Hypertrophy, Fibrosis, and Apoptosis

**DOI:** 10.3390/ijms17050749

**Published:** 2016-05-18

**Authors:** Juan Wang, Oi Wah Liew, Arthur Mark Richards, Yei-Tsung Chen

**Affiliations:** 1 Cardiovascular Research Institute, National University Health System, Singapore 119228, Singapore; mdcwaju@nus.edu.sg (J.W.); mdclow@nus.edu.sg (O.W.L.); arthur_mark_richards@nuhs.edu.sg (A.M.R.); 2 Department of Medicine, Yong Loo Lin School of Medicine, National University of Singapore, Singapore 117597, Singapore; 3 Cardiac Department, National University Health System, Singapore 119228, Singapore; 4 Christchurch Heart Institute, University of Otago, Christchurch 8014, New Zealand

**Keywords:** microRNA, hypertrophy, fibrosis, apoptosis

## Abstract

MicroRNAs (miRNAs) are non-coding RNAs that play essential roles in modulating the gene expression in almost all biological events. In the past decade, the involvement of miRNAs in various cardiovascular disorders has been explored in numerous *in vitro* and *in vivo* studies. In this paper, studies focused upon the discovery of miRNAs, their target genes, and functionality are reviewed. The selected miRNAs discussed herein have regulatory effects on target gene expression as demonstrated by miRNA/3′ end untranslated region (3′UTR) interaction assay and/or gain/loss-of-function approaches. The listed miRNA entities are categorized according to the biological relevance of their target genes in relation to three cardiovascular pathologies, namely cardiac hypertrophy, fibrosis, and apoptosis. Furthermore, comparison across 86 studies identified several candidate miRNAs that might be of particular importance in the ontogenesis of cardiovascular diseases as they modulate the expression of clusters of target genes involved in the progression of multiple adverse cardiovascular events. This review illustrates the involvement of miRNAs in diverse biological signaling pathways and provides an overview of current understanding of, and progress of research into, of the roles of miRNAs in cardiovascular health and disease.

## 1. Introduction

MicroRNAs (miRNAs) are small non-coding RNAs that have emerged as essential regulators for almost all aspects of intracellular signaling pathways in eukaryotic cells. MiRNAs originate from long primary transcripts (pri-miRNA) and are subsequently cleaved by the nuclear ribonuclease, Drosha, to form microRNA precursors (pre-miRNA) of approximately 70-nucleotides. Pre-miRNAs are exported to the cytoplasm and further processed into approximately 22-nucleotide double-stranded miRNAs by the endonuclease, Dicer. One of the strands, namely the guide strand or mature miRNA, binds to Argonaute proteins within the RNA-induced silencing complex (RISC). The RISC utilizes the guide strand to target the 3′ end untranslated regions (3′UTR) of gene transcripts by Watson-Crick base pairing. The action of miRNAs usually results in downregulation of their target genes by triggering messenger RNA degradation or by inhibiting the translational machinery [1]. The importance of miRNAs in the cardiovascular system is demonstrated by the profound structural and functional abnormalities observed in cardiac-specific Dicer deletion in animal models [2,3,4]. For the past decade, studies using various cellular and animal disease and transgenic models have allowed identification of miRNAs and their target genes, in various cardiovascular disease states. By definition, cardiac remodeling is a dynamic and adaptive process in the normal state (physiological remodeling) or in the course of disease progression (pathological remodeling) where the heart alters its structure to regain hemodynamic homeostasis against mechanical stretch or extrinsic stimulation by neurohormones or cytokines. In the diseased state, however, pathological cardiac remodeling is often triggered by conditions that lead to pressure or volume overload, such as myocardial infarction, valve stenosis or regurgitation, hypertension, myocarditis, and a broad range of familial and acquired cardiomyopathies [5]. Neurohormonal derangement such as prolonged stimulation from over-activated renin-angiotensin-aldosterone (RAAS) and sympathetic nervous (SNS) systems are triggered by cardiac injury and/or overload and also contribute directly to adverse cardiac remodeling. The mechanisms of pathological cardiac remodeling involve complex cellular and molecular signaling cascades that regulate myocyte growth, hypertrophy, cardiac fibrosis, necrosis, apoptosis, electrical conduction, and metabolic homeostasis. To date, the involvement of miRNAs in cardiac dysfunction are best characterized in hypertrophy, fibrosis, and apoptosis.

The roles of miRNAs in regulating the expression of genes related to cardiovascular diseases have been investigated in numerous cellular and animal models and have been reviewed extensively [6,7,8]. However, validation of specific miRNA target genes and identification of the affected signaling pathways have not yet been extensively completed and discussed. Identification of target mRNAs for specific miRNAs is the crucial first step for elucidating the physiological functions of miRNAs. Since most miRNAs are only partially complementary to their target sites, *in silico* prediction on the basis of sequence homology alone is not sufficient for identifying miRNA targets in real biological systems. Experimental validation is required in determining physiologically relevant gene targets. In this review, the regulatory effects of microRNAs in cardiac remodeling, particularly cardiac hypertrophy, fibrosis, and apoptosis are reviewed. Modulation of the expression of target genes by the miRNAs selected for this review has been demonstrated by miRNA/3′UTR mRNA interaction assays, and/or by gain- or loss-of-function assays in cardiac-derived cell lineages.

## 2. Hypertrophy-Related miRNAs

Biomechanical stress and other pathological stimuli potentially trigger cardiac remodeling manifested as cardiac hypertrophy with an increase in myocyte size and myofibrillar volume, but not myocyte cell number facilitating sustained cardiac output in the face of functional insult. However, whilst such an adaptive mechanism is beneficial in the short term, prolonged hypertrophy has potentially disastrous consequences leading ultimately to heart failure and death. Studies profiling dysregulated miRNA expression in animal models of cardiac hypertrophy induced by thoracic-aortic banding or via constitutively activated calcineurin signaling have revealed both up- and down-regulated miRNAs. Functional analyses of these dysregulated miRNAs have demonstrated that miRNAs may exert either positive or negative regulatory effects on cardiac hypertrophic pathways.

The role of one of the most abundant cardiac miRNAs, miR-1, in hypertrophy was suggested by the inverse relationship between its expression and progression of cardiac hypertrophy in a transverse aortic constriction (TAC) rodent model. Gain-of-function miRNA studies in neonatal myocyte culture demonstrated downregulation of several hypertrophic target genes of miR-1 predicted *in silico*, including Ras GTPase-activating protein (RasGAP), cyclin-dependent kinase 9 (Cdk9), Ras homolog enriched in brain (Rheb), and fibronectin [9]. Further studies demonstrate that miR-1 prevents cardiac hypertrophy by suppressing heart and neural crest derivatives expressed 2 (Hand2), and by inhibiting the activity of insulin-like growth factor (Igf1) and extracellular matrix remodeling factor, twinfilin 1 (Twf1) [10,11]. miR-101 was shown to regulate the expression of ras-related protein-1A (Rab1A) in the TAC-induced hypertrophy rodent model and in angiotensin II induced cellular hypertrophy. Antagonism of miR-101 attenuated the hypertrophic phenotype in both models [12]. The role of miR-133 in regulating cardiac hypertrophy was demonstrated by the induction of cardiac hypertrophy in miR-133 null mice. Molecular investigations revealed that miR-133 targets multiple anti-hypertrophic genes, including guanosine triphosphate-guanosine diphosphate (GDP-GTP) exchange protein, Rhoa, signal transduction kinase cell division control protein 42 (Cdc42), and the nuclear factor, negative elongation factor complex member A (Nelfa/Whsc2) [13]. miR-145 negatively regulates the expression of GATA binding protein 6 (GATA6) and subsequently attenuates hypertrophy in isoproterenol-induced cellular hypertrophy models [14]. Cardiomyocyte hypertrophy induced by high glucose is modulated by miR-150 via its regulatory effect on the expression of the transcriptional co-activator, p300 [15]. Downregulation of miR-185 in a TAC-induced hypertrophy rodent model was found to produce anti-hypertrophic effects by targeting multiple genes in calcium signaling pathways, including calcium/calmodulin-dependent protein kinase II δ (Camk2d), sodium/calcium exchanger 1 (Ncx1), nuclear factor of activated T cells, cytoplasmic, and calcineurin dependent 3 (Nfatc3). miR-185 also attenuates endothelin 1-induced hypertrophy in neonatal rat myocytes, as evidenced by reductions in cellular size and expression of hypertrophic markers [16]. miR-223 antagonizes hypertrophy, induced by endothelin-1 or TAC, through targeting of cardiac troponin I (cTnI)-interacting kinase (Tnni3k) [17]. In murine models, miR-26 inhibits expression of its predicted target gene, GATA4, in TAC-induced mouse cardiac hypertrophy [18]. Similarly, down-regulation of miR-34a was observed in angiotensin II-induced hypertrophy in myocardial cells resulting in upregulation of the expression of an autophagy related gene, Agt9a [19]. Another anti-hypertrophic miRNA, miR-378, controls cardiac hypertrophy in the TAC model via repression of mitogen activated protein kinase (MAPK) signaling by targeting Mapk1, insulin-like growth factor receptor 1 (Igf1r), growth factor receptor-bound protein 2 (Grb2), and kinase suppressor of ras 1 (Ksr1) [20]. miR-9 opposes cardiac hypertrophy by targeting myocardin and reducing expression of nuclear factor of activated T cells c3 (Nfatc3). Overexpression of miR-9 suppresses cardiac hypertrophy in animal models [21]. Similarly, miR-98 was demonstrated to be an anti-hypertrophic miRNA by targeting Cyclin D2 [22].

**Figure 1:**
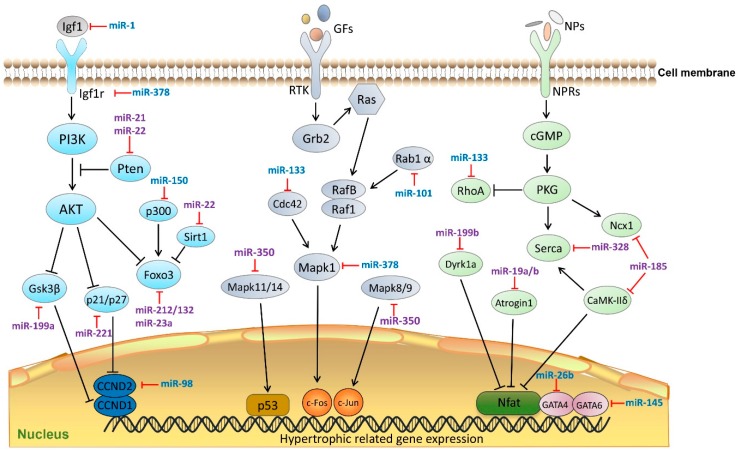
MicroRNAs (miRNAs) in cardiac hypertrophic pathways. MiRNAs regulate cardiac hypertrophy by targeting key components in three Kyoto Encyclopedia of Genes and Genomes (KEGG) signaling pathways (PI3K-AKT, MAPK, and cGMP-PKG). MiRNAs colored in blue indicate anti-hypertrophic function, miRNAs colored in purple indicate pro-hypertrophic function. Igf1, insulin-like growth factor 1; GFs, growth factors; NPs, natriuretic peptides; Igfr1, Insulin-like growth factor 1 receptor; RTK, receptor tyrosine kinases; NPRs, natriuretic peptide receptors; PI3K, phosphatidylinositol 3 kinase; Pten, phosphatase and tensin homolog; AKT, protein kinase B; Gsk3β, glycogen synthase kinase 3 β; p300, E1A binding protein p300; Foxo3, Forkhead box O 3; Sirt1, Sirtuin 1; CCND1, Cyclin D1; CCND2, Cyclin D2; Ras, small G-protein; Grn2, growth factor receptor-binding protein 2; Rab1α, Ras-related protein Rab 1 α; RafB/Raf1, B-Raf proto-oncogene serine/threonine-protein kinase; Cdc42, cell division cycle 42; Mapk1, Mitogen-activated protein kinase 1; Mapk11/14, mitogen-activated protein kinase 11/14; Mapk 8/9, mitogen-activated protein kinase 8/9; p53, tumor protein p53; c-Fos, proto-oncogene protein; c-Jun, transcription factor AP-1; cGMP, cyclic guanosine monophosphate; PKG, protein kinase G; Rhoa, Ras homolog family member A; Ncx1, codium/calcium exchanger 1; Serca, Sarco/endoplasmic reticulum Ca^2+^-ATPase; Dyrk1a, dual specificity tyrosine-(Y)-phosphorylation regulated kinase 1A; CaMK-IIδ, calcium/calmodulin-dependent protein kinase II δ; Nfat, nuclear factor of activated T-cells; GATA6, GATA binding protein 6; GATA4, GATA binding protein 6.

In contrast, other miRNAs are pro-hypertrophic. For instance, miR-155 has been reported to play a role in hypertrophy and cardiac remodeling by targeting tumor protein p53-induced nuclear protein (Tp53inp1) [23]. The miR-19a/b family directly targets the anti-hypertrophic genes atrogin 1 and muscle ring finger protein 1 (Murf1) with subsequent activation of calcineurin/nuclear factor of activated T cells (NFAT) signaling. Overexpression of miR-19a/b induces hypertrophy in rat neonatal cardiomyocytes [24]. miR-199a impairs autophagy and induces cardiac hypertrophy through activation of mTOR signaling [25,26]. miR-199b plays an important role in the activation of calcineurin/NFAT signaling by targeting Nfat kinase dual-specificity tyrosin-(Y)-phosphorylation regulated kinase 1a (Dyrk1a). Antagonism of miR-199b in a mouse heart failure model significantly suppressed the progression of cardiac hypertrophy and fibrosis [27]. Another cluster of miRNAs, namely miR-208a and miR-208b are known to be pro-hypertrophic through targeting of negative regulators of hypertrophy including thyroid hormone-associated protein 1 (Thrap1) and myostatin 2 [28]. Furthermore, one of the most well-known cardiovascular disease related miRNAs, miR-21, is thought to target phosphatase and tensin homolog (Pten) and subsequently modulate the activation of the AKT/mTOR pathway, resulting in hypertrophy and fibrosis [29]. Interestingly, its passenger strand, miR-21-3p also exerts hypertrophic effects by targeting histone deacetylase-8 (Hdac8), sorbin and SH3 domain-containing protein 2 (Sorbs2), and PDZ-LIM domain 5 (Pdlim5) [30,31]. By targeting the anti-hypertrophic Forkhead box O3 (Foxo3) transcription factor, miR-212/132 and miR-23a activate pro-hypertrophic calcineurin/NFAT signaling [32,33]. miR-23a may also regulate the expression of lysophosphatidic acid receptor 1 (Lpa1) and induce cardiomyocyte hypertrophy [34]. Several studies support a role for miR-22 in cardiac hypertrophy via targeting Pten, Sirtuin 1 (Sirt1), and histone deacetylase 4 (Hdac4) as evidenced by results from transgenic mouse models and multiple induced-hypertrophic cellular platforms [35,36,37]. miR-221 is thought to play a role in the development of cardiac hypertrophy in heart disease via targeting of cyclin-dependent kinase inhibitor 1B (p27) [17]. miR-27b targets the anti-hypertrophic transcription factor, peroxisome proliferator-activated receptor γ (Pparγ) [38]. Cardiac hypertrophy induced by angiotensin II both *in vitro* and *in vivo*, is mediated by downregulation of beclin 1 by miR-30a [39]. The passenger strand, miR-30a-3p is also involved in autophagy and cardiac hypertrophy via targeting of X-box binding protein 1 (Xbp1), a stress response transcription factor [40]. In an animal model of pressure overload, the expression of the key calcium-transporting ATPase responsible for Ca^2+^ re-uptake, Sarco/endoplasmic reticulum Ca^2+^-ATPase 2a (ATP2a2, also known as Serca2a), is targeted by miR-328 and subsequently resulting in activation of calcineurin/NFAT signaling, one of the signature pathways leading to hypertrophy [41]. In the rat TAC-induced hypertrophic model miR-350 targets Mapk11/14 and Mapk8/9. Upregulation of miR-350 attenuated the inhibitory effect exerted by p38 and Jun amino-terminal kinases (JNK) pathways on calcineurin/NFAT signaling leading to cardiomyocyte hypertrophy and apoptosis [42]. Figure 1 illustrates the interactions between known miRNAs in three classical hypertrophic signaling pathways, namely phosphatidylinositol 3 kinase-protein kinase B (PI3K-AKT), mitogen-activated protein kinase (MAPK) and cyclin guanosine monophosphate-dependent protein kinase G (cGMP-PKG).

Table 1 summarizes miRNAs that have been implicated in cardiac hypertrophy and their target genes, as well as the experimental platforms and methods used in each studies.

## 3. miRNAs in Cardiac Fibrosis

Cardiac fibrosis is a common phenotype, found in several cardiac diseases including myocardial infarction and heart failure, characterized by the adverse accumulation of collagens and other extracellular matrix (ECM) proteins. Early work from Thum *et al.* [48] demonstrated that miR-21 promotes cardiac fibrosis by targeting extracellular regulated kinase inhibitor sprouty homolog 1 (Spry1) with stimulation of MAPK signaling in cardiac fibroblasts. In a model of myocardial ischemia-reperfusion, miR-21 was found to target Pten and subsequently leading to an increase in matrix metalloprotease 2 (Mmp2). In line with this finding, antagonism of miR-21 results in an increase of Pten in cardiac fibroblasts [49]. In a pioneer study by Olson’s group, expression of miR-29 family members (miR-29a, miR-29b and miR-29c) negatively correlated with expression of genes involved in ECM production and fibrosis after experimental myocardial infarction. Furthermore, the miR-29 family regulates the expression of pro-fibrotic genes, including extracellular matrix genes elastin [50], fibrillin 1 (Fbn1), collagen type I, α 1 and 2 (Col1α1, Col1α2) and collagen type III, α 1 (Col3α1) [51]. In addition, miR-29b targeting Tgfβ1 to play a role in transforming growth factor β (TGFβ)/SMAD3 signaling [52]. Several other miRNAs have been identified as also targeting collagens and TGFβ signaling to play a role in fibrogenesis. For instance, Let-7i and miR-26a attenuate collagen deposition and exert their function through targeting Col1α2 and Col1α1, respectively [23,53,54]. The role of miR-133a in myocardial fibrosis and electrical repolarization in pressure-overloaded adult hearts may reflect its regulatory effects on the expression of Col1α1, Serca2a and calcineurin [55,56]. Recently, miR-101and miR-101a were found to negatively regulate TGFβ signaling through targeting of Tgfβ receptor type 1 (Tgfβr1) and c-Fos. Cardiac fibrosis is significantly suppressed by upregulation of miR-101a in the rat myocardial infarction model [57,58]. miR-125b is pro-fibrotic via targeting of apelin, one of the key fibrogenesis repressors in the heart [59]. Wang *et al.* [60] showed that miR-24 could interfere with TGFβ signaling by targeting the pro-protein convertase, furin, and subsequently downregulate the level of TGFβ in cardiac fibroblasts. By targeting connective tissue growth factor (Ctgf), miR-133 and miR-30 are suggested to be important for collagen synthesis [61].

**Figure 2:**
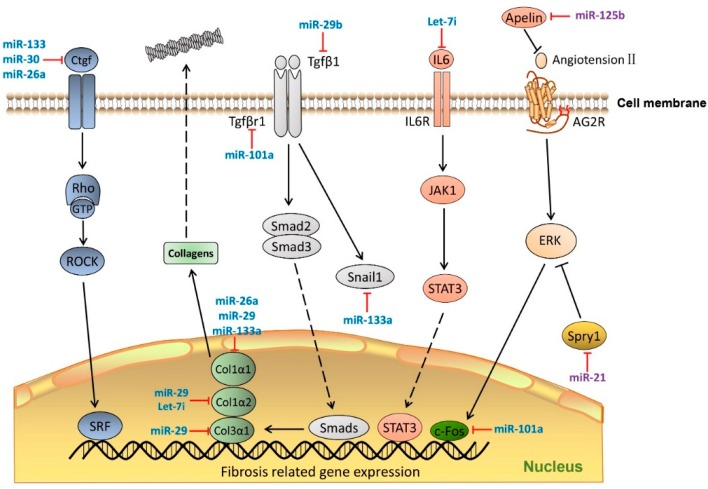
MiRNAs in cardiac fibrosis pathway. MiRNAs regulate cardiac fibrosis process by targeting the key molecules that mediate transcription of ECM genes and TGFβ signaling. MiRNAs colored in blue indicate anti-fibrosis function, miRNAs colored in purple indicate pro-fibrosis function. Dashed lines indicate translocation of molecules from cytoplasm to nucleus. Ctgf, connective tissue growth factor; Rho-GTP, Rho-GTPase-activating protein; ROCK, Rho associated coiled-coil containing protein kinase; SRF, Serum response factor; Col1a1, Collagen, type 1 α 1; COL1α2, collagen, type 1 α 2; COL3α1, collagen, type 3 α 1; Tgfβ1, transforming growth factor β 1; TgfβR1, transforming growth factor β receptor 1; Smad2/3, SMAD family member 2/3; Snail1, snail family zinc finger 1; IL6, Interleukin 6; IL6R, interleukin 6 receptor; JAK1, Janus kinase 1; STAT3, signal transducer and activator of transcription 3; c-Fos, FBJ murine osteosarcoma viral oncogene homolog; Spry1, sprouty homolog 1. Dash line arrows indicate the relocation of proteins; solid lines arrows indicate signaling cascades.

Figure 2 provides a schematic representation of the involvement of known miRNAs in classical fibrogenesis signaling pathways.

Table 2 lists miRNAs related to cardiac fibrogenesis, their target genes, and the experimental platforms used in studies.

## 4. miRNAs in Cardiomyocyte Apoptosis

Apoptosis is programmed cell death which normally ensures proper functional and metabolic homeostasis in multicellular organisms. As with other eukaryotic cells, apoptosis in cardiomyocytes may be triggered by the activation of two main pathways, an extrinsic pathway that is mediated by death receptors, and an intrinsic pathway that involves mitochondrial permeability/transmembrane potential. Both pathways are known to activate initiator caspases (caspase 8, 9, 10) and finally the apoptosis executioner, caspase 3 (Casp3) [63]. Results from one recent study demonstrated that miR-133 and miR-874 negatively regulate the expression of Casp3 and Casp8, respectively, and protect cardiomyocytes from cell death induced by oxidative stress [64]. MiR-133 also regulates expression of Casp9 with subsequent reduction in Casp3 levels in nicotine-induced cell death [65]. Casp3 is also targeted by miR-378 with consequent attenuation of ischemia-induced apoptosis [66]. Srivastava and colleagues used an ischemia-reperfusion injury rat model to show the beneficial effect of ischemic post-conditioning may be mediated by the regulatory effect of miR-133a on Casp9 [67]. In addition to caspases, miRNAs have also been shown to target the B-cell lymphoma 2 (Bcl2) family within the intrinsic mitochondrial apoptosis pathway. Functionally, these miRNAs could be divided into anti-apoptosis and pro-apoptosis sub-families. At present, miR-1, miR-15b, miR-30b, miR-34a, and miR-497 are reported to promote cardiomyocyte cell death through repression of Bcl2 (anti-apoptotic) gene expression whereas miR-149 and miR-24 repress apoptosis by targeting the pro-apoptotic genes Puma and Bim [68,69,70,71,72,73,74].

In addition to targeting Bcl2 or caspase families, miRNAs were reported to target various components of the upstream apoptosis-related signaling cascades. For instance, miR-1 mediates apoptosis of H9c2 cells induced by high glucose, by suppressing the expression of anti-apoptotic genes such as insulin-like growth factor (Igf1). In a transgenic mouse overexpression of miR-1 exacerbated cardiac apoptosis secondary to ischemia-reperfusion. Further mechanistic studies revealed that protein kinase C ε (Pkcε) and heat shock protein 60 (Hsp60) are targets for miR-1 [75,76]. Similarly, miR-320 promotes apoptosis of cardiomyocytes by reducing the level of heat shock protein 20 (Hsp20) [77].

In contrast to be the positive regulators of cardiac apoptosis, several miRNAs such as miR-21, miR-199a, and miR-30 have been reported to antagonize apoptosis of cardiac cells. Multiple lines of evidence demonstrate that miR-21 attenuates cardiac apoptosis secondary to oxidative stress-induced cardiac apoptosis by suppressing expression of programmed cell death 4 (Pdcd4) [78,79]. The protective role of miR-21 against cardiac apoptosis is further substantiated by the observation that during hydrogen peroxide-induced injury in cardiac myocytes knockdown of miR-21 induces, while upregulation of miR-21 protects against, cell death [79]. MiR-21 also suppresses apoptosis induced by hypoxia through targeting of the PTEN/AKT pathway [80]. Like miR-21, miR-199a regulates the expression of hypoxia-inducible factor 1 α (Hif1α) and a class II histone deacetylase, sirtuin 1 (Sirt1). Overexpression of miR-199a under hypoxic conditions downregulates Hif1α and Sirt1 with subsequent suppression of p53 and attenuation of hypoxia-induced cardiomyocyte apoptosis [81]. Interestingly, one study demonstrated that p53 is also targeted by the miR-30 family (miR-30a-e). In apoptosis induced by oxidative stress, downregulation of miR-30 upregulates p53 and dynamin-related protein 1 (Drp1), an initiator of mitochondrial fission, finally culminating in apoptosis [82]. The miR-30 family was found to mediate doxorubicin-induced apoptosis in adult rat cardiomyocytes. Additional experiments demonstrated that miR-30 targets multiple genes in the β-adrenergic signaling pathway including β1 and β2 adrenoceptors and G protein α i subunit (G_iα_2) [83]. Several other miRNAs have been demonstrated to play critical roles in hypoxia-induced apoptosis in cardiomyocytes. Among them, miR-100, miR-101, miR-132, miR-15b, miR-20a, and miR-92a were found to enhance apoptosis by targeting negative regulators/factors in the intrinsic apoptosis pathway [69,81,84,85,86,87,88]. In contrast, miR-133a, miR-138, and miR-146b were demonstrated to attenuate hypoxia-induced apoptosis by targeting different upstream signaling pathways [80,89,90,91,92]. Numerous other miRNAs have been demonstrated to exert pro-apoptotic or anti-apoptotic effects by targeting various signaling networks upstream of the intrinsic apoptosis pathway.

**Figure 3:**
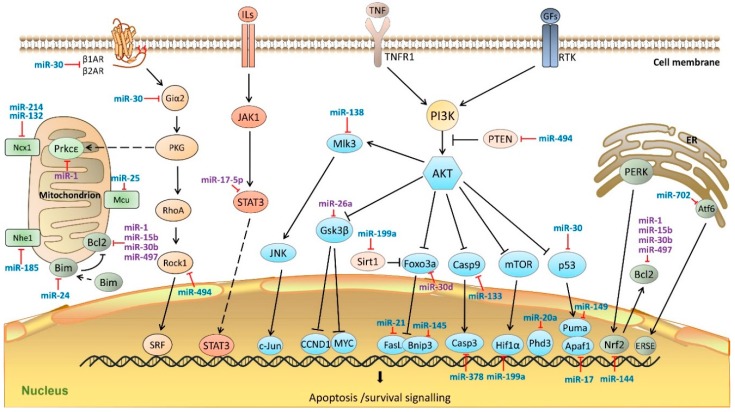
MiRNAs in cardiomyocyte apoptosis pathway. MiRNAs regulate cardiac apoptotic processes by targeting key molecules in mitochondria, endoplasmic reticulum, as well as, JAK-STAT3 and PI3K/AKT pathways. MiRNAs colored in blue indicate the anti-apoptosis functions, miRNAs colored in purple indicate pro-apoptosis functions. β1AR, adrenoceptor β 1; β2AR, adrenoceptor β 2; Giα2, G protein α i subunit; PKG, cGMP-dependent protein kinase; RhoA, Ras homolog family member A; STAT3, Signal transducer and activator of transcription 3; Rock1, Rho associated coiled-coil containing protein kinase 1; SRF, serum response factor; Ncx1, sodium/calcium exchanger 1; Nhe1, Na^+^/H^+^ exchanger 1; Prkcε, protein kinase C ε; Mcu, mitochondrial calcium uniporter; Bcl2, B-cell CLL/lymphoma 2; Bim, Bcl2 like 11; ILs, Interleukins; JAK1, Janus kinase 1; STAT3, signal transducer and activator of transcription 3; c-Fos, FBJ murine osteosarcoma viral oncogene homolog; TNF, tumor necrosis factor; TNFR1, tumor necrosis factor receptor 1; GFs, growth factors; RTK, receptor tyrosine kinases; PI3K, phosphatidylinositol 3 kinase; Pten, phosphatase and tensin homolog; AKT, Protein kinase B; Mlk3, mitogen-activated protein kinase 11; JNK, mitogen-activated protein kinase 8; c-Jun, transcription factor AP-1-like; Gsk3β, glycogen synthase kinase 3 β; CCND1, cyclin D1; MYC, myelocytomatosis oncogene; Foxo3a, Forkhead box O 3a; Sirt1, Sirtuin 1; FasL, Fas ligand; Bnip3, Bcl2/adenovirus E1B 19 kDa interacting protein 3; Casp9, caspase 9; Casp3, caspase 3; m-TOR, mechanistic target of rapamycin; Hif1α, hypoxia-inducible factor 1, α subunit; Phd3, Egl-9 family hypoxia-inducible factor 3; p53, Tumor protein p53; Puma, Bcl2 binding component 3; Apaf1, Apoptotic peptidase activating factor 1; PERK, eukaryotic translation initiation factor 2 α kinase 3; Atf6, activating transcription factor 6; Nrf2, nuclear factor, erythroid 2 like 2; ERSE, ER stress response element; ER, endoplasmic reticulum. Dash line arrows indicate the relocation of proteins; solid line arrows indicate signaling cascades.

Figure 3 illustrates the involvement of known miRNAs in classical signaling pathways regulating apoptosis.

Apoptosis is a complex and highly sophisticated series of physiological events that can be divided into three phases, namely initiation, propagation, and execution [93]. Several microRNAs reviewed in this section were found to directly target the key molecules involved in different apoptotic phases. Through their modulating influence on apoptotic signaling pathways, miRNAs can exert pro-apoptotic or anti-apoptotic effects and, thus, can have a profound influence on cardiac cell survival. For instance, miR-132, miR-145, miR-214, and miR-25 were demonstrated to target the intracellular calcium regulating pathway and subsequently inhibit the initiation of apoptosis [86,94,95,96]. miR-24 was found to negatively regulate expression of the pro-apoptosis Bcl-2 family protein, Bim, and represses the initiation of mitochondrial and ER stress-induced apoptosis [74]. Conversely, miR-1 and miR-30b were shown to directly suppress the expression of Bcl-2 and facilitate the initiation of apoptotic processes in cardiomyocytes in various disease models [70,76]. miR-133 and miR-17 were reported to regulate the expression of Casp9 and apoptotic protease-activating factor 1 (Apaf-1), respectively, the key molecules in apoptosis propagation [65,97]. MiR-378 was demonstrated to suppress the expression of Casp3 and block the entry to the execution phase in cardiomyocytes [66]. Known cardiac apoptosis-related miRNAs and their target genes, as well as the experimental platforms and methods used in studies are summarized in Table 3.

## 5. Future Perspectives

MiRNAs have emerged as vital post-transcriptional regulators involved in almost all biological processes ranging from development, such as stem cell differentiation, to maintenance of cell and tissue integrity for multiple physiological functions. The roles of miRNAs in cardiovascular pathophysiological events and the therapeutic potential of targeting specific miRNA in ameliorating progression towards cardiac hypertrophy, fibrosis, and apoptosis are under ongoing exploration in various *in vitro* and *in vivo* disease models. Clearly, as discussed in this review, each cardiac pathophysiological event is regulated by multiple microRNAs. As illustrated in Table 4, a number of known microRNAs are not functionally restricted but have been shown to participate in multiple aspects of cardiac pathophysiology. Since the predominant cardiovascular diseases such as heart failure or hypertension result from complex pathophysiology the elucidation of the roles of multifunctional disease-related miRNAs will provide targets for developing therapeutics able to simultaneously suppress multiple disease-mediating pathological pathways. Recent technological advances in unraveling the complexity of miRNA biology and function, particularly in relation to the cardiovascular system, is crucial for accurate translation of knowledge to alter specific disease-causing signaling cascades, making the development of miRNA-mediated clinical intervention a reachable reality in the near future. However, the challenge remains particularly in integrating laboratory and clinical findings to design innovative research platforms to translate research findings into clinical applications such as biomarkers and/or therapeutic intervention.

Cardiovascular diseases, such as heart failure (HF), are complex with contributions from various genomic, genetic, and environmental factors. To date, although numerous genome-wide studies have revealed differential expression patterns of miRNAs in HF compared to controls, signature miRNA profiles for HF have yet to be definitively identified. By compiling reports from different HF cohorts it is apparent a number of apoptosis-related miRNAs display consistent differential expression patterns in various biological specimens including serum, plasma and cardiac tissue. For instance, miR-1 and miR-21 were originally demonstrated to play roles in cardiac remodeling and apoptosis-related signaling in cellular and animal models. A later genome-wide miRNA study revealed that these two miRNAs together with several other cardiac hypertrophy-related miRNA (miR-23) and apoptosis-related miRNAs (miR-195 and miR-199), were differentially expressed in myocardial biopsies from HF patients compared with controls, suggesting the pervasive involvement of these miRNAs in the pathology of HF [117]. Similarly, another cluster of apoptosis-related miRNAs (miR-145, miR-214, miR-378, and miR-7), as well as miR-1 were found differentially expressed in HF cardiac tissues in another study [118]. One recent HF cohort study revealed differential expression of a cluster of hypertrophy-related miRNAs (miR-101 and miR-22), a cluster of apoptosis-related miRNAs (miR-17, miR-30a, and miR-92a) and miR-21 in serum [119]. Further comparisons of 10 selected miRNAs listed in Table 4 with data originating from 9 HF cohorts [117,118,119,120,121,122,123,124] revealed that most of these multifunctional miRNAs are reported as dysregulated in more than one cohort study, suggesting clinical significance. Amongst these, miR-1 was shown to be differentially expressed in four cohorts [117,118,122,124], and miR-21 was reported to differentially expressed in two cohort studies [117,119]. Comparative evaluation across different data bases, particularly in multiple sample types, investigative models, patient cohorts and disease phenotypes, is necessary to shortlist candidate miRNAs for development of putative therapeutic applications in the future.

Over the past decade the essential roles of miRNAs in the biology and cellular dysfunction of cardiovascular pathologies have been revealed at an increasing pace. In this article, by compiling miRNAs associated with cardiac hypertrophy, fibrosis, and apoptosis, we hope to bring insights for the roles of miRNAs in cardiac remodeling pathways to the readers. This body of information provides the foundation for further innovative research towards development of miRNA-based diagnostic methods and therapeutics.

## Figures and Tables

**Table 1:** Summary of reported miRNAs and their targets in cardiac hypertrophy.

miRNAs	Targets		miRNA–mRNA Interaction		Platforms Remarks	References
	mRNA	Signaling Pathway	Luciferase Assay	Gain/Loss of Function		
***Anti-hypertrophic***						
miR-1	Fbln2	ECM	+	+	AAB rat	[11]
	Twf1	ECM	+	+	NRCMs	[10]
	Igf1	PI3K-Akt	+	+	TAC mouse and AAC rat	[43,44]
	Fabp3	PPAR	+	+	NMCMs and TAC mouse	[45]
miR-101	Rab1a	MAPKK	+	+	TAAC rat	[12]
miR-133	RhoA	cGMP-PKG	+	+	NMCMs and TAC mouse	[13]
	Cdc42	MAPK	+	+	NMCMs and TAC mouse	
	Nelfa/Whsc2	Transcription	+	+	NMCMs and TAC mouse	
miR-145	GATA6	cGMP-PKG	+	+	NRCMs and TAC mouse	[14]
miR-150	p300	FoxO	+	+	NRCMs	[15]
miR-185	Camk2d	Calcium	+	+	NRVMs and TAC mouse	[16]
	Ncx1	cGMP-PKG	+	+	NRVMs and TAC mouse	
	Nfatc3	cGMP-PKG	+	+	NRVMs and TAC mouse	
miR-223	Tni3k		+	+	NRCMs and TAC mouse	[46]
miR-26b	GATA4	cGMP-PKG	+	+	NRCMs and TAC mouse	[18]
miR-30-3p	Xbp1	VEGF	+	+	H9c2 cells and AAC rat	[40]
miR-34a	Atg9a	Autophagy	+	−	NRCMs and TAAC rat	[19]
miR-378	Mapk1	MAPK	+	+	NRCMs and TAC mouse	[20]
	Igf1r	MAPK	+	+	NRCMs and TAC mouse	
	Grb2	MAPK	+	+	NRCMs and TAC mouse	
	Ksr1	MAPK	+	+	NRCMs and TAC mouse	
miR-9	Myocardin	cGMP-PKG	+	−	NRCMs	[21]
miR-98	Cyclin D2	PI3K-AKT	+	−	NRCMs	[22]
***Pro-hypertrophic***						
miR-155	Tp53inp1	p53	+	+	NMCFs and AMI mouse	[23]
miR-199a	Gsk3β	PI3K-AKT	+	+	NRCMs and TG mouse	[25]
miR-199b	Dyrk1a	calcineurin/NFAT	+	+	NRCMs and TAC mouse	[27]
miR-19a/b	Atrogin1	calcineurin/NFAT	+	+	NRCMs and TAC mouse	[24]
	Murf1	PKC	+	+	NRCMs and TAC mouse	
miR-208a	Thrap1	Thyroid hormone	+	+	NRCMs, TAC and TG mouse	[28]
	Myostatin	Cell growth/Differentation	+	+	NRCMs, TAC and TG mouse	
miR-21	Pten	PI3K-AKT	+	−	Human glomerular mesangial cells	[47]
miR-21-3p	Hdac8	AKT/GSK3β	+	+	TAC mouse	[30]
miR-212/132 family	Foxo3	PI3K-Akt	+	+	H9c2 cells and TAC mouse	[32]
miR-22	Sirt1	AMPK	+	+	NRCMs, miR-22 KO mouse	[36]
	Hdac4	AMPK	+	+	NRCMs, miR-22 KO mouse	
	Pten	PI3K-AKT	+	+	NRCMs	[35]
miR-221	p27	PI3K-AKT	+	+	NRCM, TAC mouse	[17]
miR-23a	Foxo3a	PI3K-AKT	+	−	NMCMs, TAC and TG mouse	[33]
	Lpa1	PI3K-AKT	+	+	NRCMs	[34]
miR-27b	Pparγ	PPAR	+	+	NRCMs, TAC and TG mouse	[38]
miR-30a	Beclin 1	Autophagy	+	+	NRCMs, TAAC rat	[39]
miR-328	Serca2a	cGMP-PKG	+	+	NRVCs, TAC and TG mouse	[41]
miR-350	Mapk11/14	MAPK	+	+	H9c2 cells and TAC rats	[42]
	Mapk8/9	MAPK	+	+	H9c2 cells and TAC rats	

Abbreviations for mRNAs: Fbln2, Fibulin 2; Twf1, Twinfilin 1; Igf1, Insulin-like growth factor 1; Fabp3, Fatty Acid Binding Protein 3; Rab1a, Ras-related protein Rab 1a; Rhoa, Ras homolog family member A; Cdc42, Cell Division Cycle 42; Nelfa/Whsc2, Wolf-Hirschhorn syndrome candidate 2 protein; GATA6, GATA binding protein 6; p300, E1A binding protein p300; Camk2d, calcium/calmodulin-dependent protein kinase II delta; Ncx1, sodium/calcium exchanger 1; Nfatc3, Nuclear factor of activated T-cells, cytoplasmic, calcineurin dependent 3; Tnni3k, Troponin I type 3 interacting kinase; GATA4, GATA binding protein 4; Xbp1, X-box binding protein 1; Atg9a, autophagy-related 9A; Mapk1, mitogen-activated protein kinase 1; Igf1r, insulin-like growth factor 1 receptor; Grb2, Growth factor receptor bound protein 2; Ksr1, kinase suppressor of ras 1; Tp53inp1, tumor protein p53 inducible nuclear protein 1; Gsk3β, glycogen synthase kinase 3 β; Dyrk1a, Dual-specificity tyrosine-(Y)-phosphorylation regulated kinase 1a; Murf1, tripartite motif-containing 63; Thrap1, Mediator complex subunit 13; Pten, phosphatase and tensin homolog; Hdac8, histone deacetylase 8; Foxo3, Forkhead box O3; Sirt1, Sirtuin 1; Hdac4, histone deacetylase 4; Foxo3a, Forkhead box O3A; Lpa1, Lysophosphatidic acid receptor 1; Pparγ, peroxisome proliferator-activated receptor γ; Serca2a, Sarco/endoplasmic reticulum Ca^2+^-ATPase 2a; Mapk11/14, mitogen-activated protein kinase 11/14; Mapk8/9, Mitogen-activated protein kinase 8/9. Abbreviations for pathway: ECM, extracellular matrix deposition; PI3K-Akt, phosphatidylinositol 3 kinase-protein kinase B; PPAR, peroxisome proliferator-activated receptor; cGMP-PKG, cyclic guanosine monophosphate-dependent protein kinase G; VEGF, vascular endothelial growth factor; Akt-Gsk3β, protein kinase B-Glycogen synthase kinase 3 β; FoxO, Forkhead box; MAPK, mitogen-activated protein kinase; p53, tumor protein p53; NFAT, nuclear factor of activated T-cells; PKC, protein kinase C; AMPK, AMP-activated protein kinase. Abbreviations for platforms: AAB rat, abdominal aortic banding rat; NRCMs, neonatal rat cardiomyocytes; TAC mouse, transverse aortic constriction mouse; ACC mouse, abdominal aortic constriction mouse; NMCMs, neonatal mouse cardiomyocytes; TAAC rat, transverse abdominal aortic constriction; NRVMs, neonatal rat ventricular myocytes; H9c2 cells, rat myoblast cells; NMCFs, Neonatal mouse cardiac fibroblasts; AMI mouse, acute myocardial infarction mouse; TG mouse, transgenic mouse; KO mouse, knockout mouse; NRVCs, neonatal rat ventricular cells. +/− indicates with or without experimental results to support the regulatory effect of microRNAs on target gene expressions respectively.

**Table 2:** Summary of reported miRNAs and their targets in cardiac fibrosis.

miRNAs	Targets		miRNA–mRNA Interaction		Platforms Remarks	References
	mRNA	Signaling Pathway	Luciferase Assay	Gain/Loss of Function		
***Anti-fibrosis***						
Let-7i	IL6	PI3K-AKT	+	+	NRCMs, NRCFs and Ang II induced mouse	[54]
	Col1α2	ECM	+	+	NRCMs, NRCFs and Ang II induced mouse	
miR-101a	c-Fos	MAPK	+	+	NRCFs and MI rat	[57]
	Tgfβr1	TGFβ	+	+	NRCFs and MI rat	[58]
miR-133, miR-30	Ctgf	TGFβ	+	+	RCMs, RCFs and Ren2 rat	[61]
miR-133a	Snai1	EMT	+	+	MEFs	[62]
	Col1α1	ECM	+	−	Ang II induced rat	[56]
miR-24	Furin	TGFβ	−	+	MCFs and MI mouse	[60]
miR-26a	Col1α1	PI3K-AKT	+	+	NRCFs, TAC and miR-26a TG mouse	[53]
	Ctgf	ECM	+	+	NRCFs, TAC and miR-26a TG mouse	
miR-29	Eln	Protein digestion/absorption	+	−	RCFs and MI mouse	[51]
	Fbn1	ERK	+	−	RCFs and MI mouse	
	Col1α1	ECM	+	+	RCFs and MI mouse	
	Col1α2	ECM	+	+	RCFs and MI mouse	
	Col3α1	ECM	+	+	RCFs and MI mouse	
miR-29b	Tgfβ1	TGFβ	−	+	MCFs	[52]
***Pro-fibrosis***						
miR-125b	Apelin	TGFβ	+	+	HCFs, TAC and Ang II induced mouse	[59]
miR-21	Spry1	ERK-MAPK	+	+	NRCFs, NRCMs, TAC and TG mouse	[48]
	Pten	PI3K-AKT	+	+	MCF and IR mouse	[49]

Abbreviations for mRNAs: IL6, Interleukin 6; Col1α2, Collagen, type I, α 2; c-Fos, FBJ murine osteosarcoma viral oncogene homolog; Tgfβr1, Transforming growth factor β receptor 1; Ctgf, connective tissue growth factor; snai1, snail family zinc finger 1; Col1α1, collagen, type 1 α 1; Eln, Elastin; Fbn1, Fibrillin 1; Col1α3, collagen, type I, α 3; Tgfβ1, transforming growth factor β 1; Spry1, sprouty homolog 1; Pten, Phosphatase and tensin homolog. Abbreviations for pathway: PI3K-AKT, phosphatidylinositol 3 kinase-rotein kinase B; MAPK, mitogen-activated protein kinase; TGF-β, transforming growth factor β; EMT, epithelial-mesenchymal transition; ERK, extracellular signal-regulated kinases; ECM, extracellular matrix. Abbreviations for platforms: NRCMs, neonatal rat cardiomyocytes NRCFs, neonatal rat cardiac fibroblasts; Ang II, angiotensin II; MI, myocardial infarction RCMs, rat ventricular myocytes; RCFs, rat cardiac fibroblasts; Ren2 rat, hypertension-induced heart failure rat model; MEFs, mouse embryonic fibroblasts; MCFs, mouse cardiac fibroblasts; HCFs, normal human cardiac fibroblasts; TG mouse, transgenic mouse; TAC mouse, transverse aortic constriction mouse. +/− indicates with or without experimental results to support the regulatory effect of microRNAs on target gene expressions respectively.

**Table 3:** Summary of reported miRNAs and their targets in cardiac apoptosis.

miRNAs	Targets		miRNA–mRNA Interaction		Platforms Remarks	References
	mRNA	Signaling Pathway	Luciferase Assay	Gain/Loss of Function		
***Anti-apoptotic***						
miR-132	Ncx1	cGMP-PKG and Calcium	+	−	NRCMs	[86]
miR-133	Casp9	ERK-MAPK	+	−	NRCMs	[65]
miR-133a	Tagln2	CASP8/CASP3	+	−	H9c2 cells	[89]
miR-138	Mlk3	TNF	+	−	H9c2 cells	[90]
miR-144	Nrf2	ROS formation	+	−	NRCMs	[98]
miR-145	Bnip3	FOXO	+	−	I/R mouse	[99]
	CamkIIδ	Calcium	+	−	NRCMs	[94]
miR-149	Puma	p53	+	−	NMCMs	[73]
miR-17	Apaf1	p53	+	−	NRCMs	[97]
miR-185	Nhe1	cAMP	+	−	NRVMs	[100]
miR-199a	Hif1α	mTOR	+	−	NRCMs	[81]
	Sirt1	AMPK	+	−	NRCMs	
miR-20a	Egln3/Phd3	HIF1	+	+	NRCMs	[87]
miR-21	Pdcd4	NFkB	+	+	NRCMs	[78]
	FasL	PI3K-AKT	+	+	NRCMs and miR-21 TG mouse	[101]
miR-214	Ncx1	Calcium	−	+	NRCM and miR-214 KO mouse	[95]
miR-24	Bim	Mitochondrial apoptosis	+	−	NMCMs	[74]
miR-25	Mcu	Mitochondrial Ca^2+^ homeostasis	+	+	H9c2 cells	[96]
miR-30	β1AR	β-adrenergic pathway	+	+	MI rat, DOX-induced HF rat, ARCM and H9c2 cells	[83]
	β2AR	β-adrenergic pathway	+	+	MI rat, DOX-induced HF rat, ARCM and H9c2 cells	
	Bnip3L/Nix	mitochondrial apoptosis	+	+	MI rat, DOX-induced HF rat, ARCM and H9c2 cells	
	G_iα2_	β-adrenergic pathway	+	+	MI rat, DOX-induced HF rat, ARCM and H9c2 cells	
	p53	p53	+	+	NRCMs	[82]
miR-378	Casp3	MAPK	+	+	H9c2 cells and AMI rat	[66]
miR-494	Pten	PI3K-AKT	+	+	miR-494 TG Mouse	[102]
	Rock1	cGMP-PKG	+	+	miR-494 TG Mouse	
	CamkIIδ	HIF1	+	+	miR-494 TG Mouse	
	Fgfr2	PI3K-AKT	+	+	miR-494 TG Mouse	
	Lif	TNF	+	+	miR-494 TG Mouse	
miR-499	Pdcd4	Mitochondrial apoptosis	+	+	NRCMs	[103]
	Pacs2	Mitochondrial apoptosis	+	+	NRCMs	
	Dyrk2	Mitochondrial apoptosis	+	+	NRCMs	
	Sox6	Cell cycle exit	+	+	P19CL6 cells and NRCMs	[104]
miR-702	Atf6	Protein process in ER	+	−	ISO treated mouse and NIH3T3 cells	[105]
miR-761	Mff	Mitochondrial apoptosis	+	−	NRCMs	[106]
miR-7a/b	Parp	DNA repair & cytoskeletal organization	+	+	H9C2 cells and I/R rat	[107]
***Pro-apoptotic***						
miR-1	Prkcε	cGMP-PKG	+	+	LNA-antimiR-1 treated mouse	[76]
	Hsp60	RNA degradation	+	+	LNA-antimiR-1 treated mouse	
	Bcl2	Mitochondrial apoptosis	+	+	H9c2 cells and I/R rat	[68]
miR-140	Mfn1	Mitochondrial fission	+	+	NRCMs	[108]
miR-146b	RNase L	NFkB	+	−	H9c2 cells	[91]
miR-15b	Bcl2	Mitochondrial apoptosis	−	+	NRVCs and I/R rat	[69]
miR-17-5p	Stat3	Jak-STAT	+	+	NRVCs and I/R rat	[109]
miR-181a	Gpx1	Mitochondrial apoptosis	+	+	H9c2 cells	[110]
miR-195	Sirt1	AMPK	+	+	NMCMs	[111]
miR-210	Aifm3	AKT/p53	+	+	H9c2 cells and NRCMs	[112]
miR-26a	Gsk3β	PI3K-AKT	+	+	NRCMs	[113]
miR-30b	Bcl2	Mitochondrial apoptosis	+	+	NRCMs	[70]
miR-30d	Foxo3a	PI3K-AKT	+	+	STZ-induced diabetic rat, NRCMs	[114]
miR-34a	Aldh2	Oxidative stress	+	+	NRCMs and MI rat	[115]
miR-497	Bcl2	Mitochondrial apoptosis	−	+	NRCMs, MI, IR mouse	[72]
miR-539	Phb2	Mitochondrial apoptosis	+	+	NMCMs and I/R mouse	[116]
miR-92a	Smad7	TGFβ	+	+	H9c2 cells	[88]

Abbreviations for mRNAs: Ncx1, sodium/calcium exchanger 1; Casp9, caspase 9; Tagln2, Transgelin 2; Mlk3, mixed-lineage protein kinase 3; Nrf2, Nuclear factor, erythroid 2 like 2; Binp3, BCL2/adenovirus E1B 19kDa interacting protein 3; CamkIIδ, calcium/calmodulin-dependent protein kinase II, δ; Puma, BCL2 binding component 3; Apaf1, apoptotic peptidase activating factor 1; Nhe1, Na+/H+ exchanger 1; Hif1α, hypoxia-inducible factor 1, α subunit; Sirt1, Sirtuin 1; Egln3/PHD3, Egl-9 family hypoxia-inducible factor 3; Pdcd4, programmed cell death 4; FasL, Fas ligand, Bim, BCL2-like protein 11; Mcu, mitochondrial calcium uniporter; β1AR, adrenoceptor β 1; β2AR, adrenoceptor β 2; Bnip3L/NIX, BCL2/adenovirus E1B interacting protein 3-like; G_iα2_, G protein α i subunit; Casp3, caspase 3; Pten, Phosphatase and tensin homolog; Rock1, Rho-associated coiled-coil containing protein kinase 1; Fgfr2, Fibroblast growth factor receptor 2; Lif, leukemia inhibitory factor; Pacs2, phosphofurin acidic cluster sorting protein 2; Dyrk2, dual-specificity tyrosine-(Y)-phosphorylation regulated kinase 2; Sox6, sex-determining region Y box 6; Atf6, activating transcription factor 6; Mff, mitochondrial fission factor; Parp, poly-(ADP-ribose) polymerase; Pkcε, protein kinase C epsilon; Hsp60, Heat shock protein 60; Bcl2, B-cell CLL/lymphoma 2; Mfn1, Mitofusin 1; RNase L, ribonuclease L; Stat3, signal transducer and activator of transcription 3; Gpx1, glutathione peroxidase 1; Gsk3β, glycogen synthase kinase-3 β; Aifm3, apoptosis inducing factor, mitochondria associated 3; Foxo3a, Forkhead box O3A; Aldh2, Aldehyde dehydrogenase 2; Phb2, Prohibitin 2; Smad7, SMAD family member 7. Abbreviations for pathway: cGMP-PKG, cyclic guanosine monophosphate-dependent protein kinase G; ERK-MAPK, extracellular signal-regulated kinases-mitogen-activated protein kinase; TNF, tumor necrosis factor; FoxO, Forkhead box O; cAMP, cyclic adenosine monophosphate; mTOR, mechanistic target of rapamycin; AMPK, 5’ adenosine monophosphate-activated protein kinase; ERS, ER stress; HIF1, Hypoxia-inducible factor 1; PI3K-Akt, phosphatidylinositol 3kinase-v-akt murine thymoma viral oncogene homolog 1; Jak-STAT, Janus kinase-signal transducer and activator of transcription; TGFβ, transforming growth factor β. Abbreviations for platforms: NRCMs, neonatal rat cardiomyocytes; H9c2 cells, rat myoblast cells; I/R, Ischemia/reperfusion; NMCMs, neonatal mouse cardiomyocytes; ARCM, adult rat cardiomyocyte; TG mouse, transgenic mouse; KO mouse, knockout mouse; AMI rat, acute myocardial infarction rat; P19CL6, mouse embryonal carcinoma; DOX, doxorubicin; HF, heart failure; ISO, isoproterenol; NIH3T3 cells, murine fibroblast cell line; NRVCs, neonatal rat ventricular cells; STZ, Streptozotocin; MI rat, myocardial infarction rat. +/− indicates with or without experimental results to support the regulatory effect of microRNAs on target gene expressions respectively.

**Table 4:** Summary of multi-functional miRNAs in cardiovascular pathophysiology.

miRNAs	Cardiac Hypertrophy	Cardiac Fibrosis	Cardiomyocyte Apoptosis
miR-1	√		√
miR-101	√	√	
miR-133	√	√	√
miR-145	√		√
miR-199a	√		√
miR-21	√	√	√
miR-24		√	√
miR-30		√	√
miR-34a	√		√
miR-378	√		√

√ indicates the involvement of microRNA in different cardiovascular pathological events.
